# Anti-Aging Effect of Dietary Fiber Compound Mediated by Guangxi Longevity Dietary Pattern on Natural Aging Mice

**DOI:** 10.3390/nu14153181

**Published:** 2022-08-03

**Authors:** Xiaohan Yu, Xiaolin Liang, Kunchen Han, Fengcui Shi, Ning Meng, Quanyang Li

**Affiliations:** 1 College of Light Industry and Food Engineering, Guangxi University, Nanning 530004, China; 1916301036@st.gxu.edu.cn (X.Y.); 1916301015@st.gxu.edu.cn (K.H.); 2016391031@st.gxu.edu.cn (F.S.); 2016401006@st.gxu.edu.cn (N.M.); 2 National Engineering Laboratory for Cereal Fermentation Technology, Jiangnan University, 1800 Lihu Road, Wuxi 214122, China; liangxiaolin@jiangnan.edu.cn; 3 Agricultural Engineering Institute, Guangxi Vocational & Technical College, Nanning 530226, China

**Keywords:** characteristic dietary fiber compound, natural aging mice, bidirectional adjustment, antioxidant, gut microbes, optimal intake parameters

## Abstract

A series of previous studies by our team has shown that the Guangxi longevity dietary pattern contributes to the improvement of human health, but the role of dietary fiber compounds (DFC) in the anti-aging of this dietary pattern has not been studied in depth. Thus, mice were fed with 5%, 15%, and 30% of the characteristic dietary fiber compound (CDFC) (compounded according to the longevity dietary pattern) for 8 weeks, and their learning memory capacity, antioxidant capacity, and inflammatory markers, as well as typical microorganisms in the intestinal tract were analyzed to investigate the anti-aging effects of the CDFC under the Guangxi longevity dietary pattern on naturally aging mice. The results showed that CDFC had a bidirectional effect on body weight regulation; increased brain, spleen, and cardiac indices, of which the medium dose was the best. Meanwhile, CDFC also had a maintenance and improvement effect on learning and memory ability in aging mice, as well as improved antioxidant capacity and reduced inflammation level. The neuronal cell necrosis in the hippocampus of mice was effectively alleviated. The expression of *Escherichia coli* and *Bacteroides* was significantly reduced, and the expression of *Bifidobacterium* and *Lactobacillus* increased. In addition, the optimal amount of CDFC added from the level of experimental animals was in a certain interval above and below 15%. The combined results indicated that CDFC mediated by the Guangxi longevity dietary pattern had significant anti-aging effects, thus theoretically proving that dietary fiber compound contributes to human longevity.

## 1. Introduction

As the global population increases and the average human lifespan increases, aging is of increasing concern to society [1]. Aging is an extremely complex biological phenomenon, and diet is one of the key factors among many complex environmental factors contributing to healthy aging [2]. Dietary fiber can reduce the risk of cardiovascular disease [3], reduce body weight in obese patients [4], modulate the immune system [5], and thus have a positive impact on human health; an important way it works is by regulating intestinal microorganisms, especially *Lactobacillus* and *Bifidobacterium*, which are considered typical of the human and animal gut and whose abundance levels are positively correlated with the health status of the host [6]. Additionally, increasing dietary fiber intake, even starting in middle age (45–65 years), has been found to reduce the risk of chronic diseases and promotes healthy aging [7]. The abundance and even the presence or absence of these probiotics are in turn closely related to the type and amount of dietary fiber consumed; therefore, it is believed that the construction of an appropriate dietary fiber intervention package and finding its optimal intake parameters is likely to be an effective way to delay aging.

Currently, most studies on dietary fiber have focused on the dietary fiber in a specific food or on a pure substance, such as pectin [8], inulin [9], etc. The beneficial effects of different dietary fibers after mixing have also been explored [10,11], but no studies have been found to report on the effects of dietary pattern-mediated characteristic dietary fiber compound (CDFC) on healthy aging. It should be clarified that the beneficial effects of individual or combinations of several dietary fibers on the human body lacking a goal-oriented approach are highly uncertain, whereas condensed dietary fiber complexes mediated by dietary patterns are likely to have better results.

Guangxi has the highest concentration of longevity in China, and previous reports suggest that longevity in this region is inextricably linked to its daily diet [12,13], which is predominantly plant foods, such as coarse grains, vegetables, and fruits, with a relatively high intake of dietary fiber [12]. The ability of the Guangxi longevity dietary pattern to reduce inflammation and improve organismal health may be closely related to its higher level of dietary fiber intake. The evaluation of the beneficial effects of dietary fiber contained in fruits, vegetables, nuts, and grains will help to dissect the relationship between diet and human health [14]. Our group previously isolated seven representative dietary fibers of fruits and vegetables from the diet of longevity population in Guangxi, and designed different dietary fiber intake methods, which initially confirmed that the addition of composite dietary fiber has a better antioxidant effect [15]. On this basis, this study intends to construct CDFC with reference to the matching way of dietary fibers in the recipes of the Guangxi longevity dietary pattern constructed in the early stage of the laboratory and to verify the anti-aging effect of CDFC by using the aging mouse model in order to provide theoretical support for the next step of defining the optimal parameters of composite dietary fibers for promoting human health and longevity.

## 2. Materials and Methods

### 2.1. Preparation of CDFC

According to the longevity dietary pattern of Guangxi constructed by the team in the early stage [16,17], combined with the survey results of centenarians [12], the dietary combination was obtained based on their dietary structure and the proportion of daily food intake, and the ingredients involved in this dietary combination were washed, dried, powdered, and sieved to form the raw material dietary mixture powder. The dietary fiber was extracted as follows: distilled water was added at a ratio of 1:20 g/mL, enzymatically digested with α-amylase at 82 °C for 1 h, pH adjusted to 10 ± 0.2 with sodium hydroxide, centrifuged (4000× *g*, 20 min) after alkaline digestion at 72 °C for 100 min, and the lower solid layer washed twice with hot water and dried and weighed, which was the insoluble dietary fiber. The supernatant was concentrated using a rotary evaporator (RE-52AA, Shanghai Yarong Biochemical Instrument Factory, Shanghai, China); 95% ethanol was added to the concentrated filtrate, and the precipitate was centrifuged overnight (8000× *g*, 15 min) The precipitate collected and freeze-dried to obtain soluble dietary fiber.

The characteristic dietary fiber compound (CDFC) was obtained by mixing soluble and insoluble dietary fiber. The composition of CDFC was determined and its total dietary fiber, fat, protein, ash, and water contents were determined according to GB 5009.88-2014, GB 5009.6-2016, GB 5009.5-2016, GB 5009.4-2016, and GB 5009.3-2016, respectively. The CDFC contained total dietary fiber (61.81%), water (6.08%), protein (2.74%), lipid (3.24%), and ash (3.88%) (dry).

### 2.2. Animals and Experimental Design

The experimental protocol and experiments were approved by the Ethics Committee of Guangxi University (Production License No: gxdxdwll01). Fifty C57BL/6J naturally aged mice (SPF, 21 months old) were purchased from the Experimental Animal Center of Guangxi Medical University (Production License No.: L20160258SCXK Gui 2020-0003). Mice were housed in conditions of 24 ± 2 °C, a relative humidity of 55 ± 5%, and a 12 h light/dark cycle, with free access to food and water. After one week of acclimatization feeding, the animals were randomly divided into 5 groups: no dietary fiber diet group (NDF), low-dose dietary fiber compound group (LDF), mid-dose dietary fiber compound group (MDF), high-dose dietary fiber compound group (HDF), and standard diet group (SD), 10 animals in each group, half male and half female, where the characteristic dietary fiber complex group feeds are based on dietary fiber-free feeds with 5%, 15%, and 30% of dietary fiber compound. The mice were weighed and recorded every two weeks for a total of 8 weeks.

### 2.3. Behavioral Tests

Open field experiment: mice were placed in a 100 cm × 100 cm × 30 cm black test chamber and allowed to explore freely for 5 min. A camera was placed on the top of the open field to record the total distance traveled, the number of times the hind limbs stood upright, and the number of squares crossed by the mice. At the end of the experiment, the urine and feces of each mouse were removed in the open field and wiped with 75% alcohol to avoid disturbing the results of the next mouse.

Morris water maze experiment: The water maze experiment was started at the end of eight weeks of intervention and lasted for five days, with a hidden platform acquisition training session on the first four days of the experiment. The positioning navigation experiment was trained four times a day; mice were allowed to enter the water from different quadrant entry points in each training session, and the timing was started while entering the water. The time for the mice to find the hidden platform was recorded, which was the escape latency. If the mice failed to find the platform within 60 s, the escape latency was recorded as 60 s for this time, and the mice were guided to the platform and allowed to stand on the platform for 10 s. The probe test was conducted on the fifth day. The test was performed by removing the platform hidden underwater, selecting the second quadrant as the entry point of the mice, and recording the evasion latency, the number of times the mice crossed the platform within 60 s, the residence time in the target quadrant, and the swimming speed of the mice.

### 2.4. Sample Collection and Processing

All mice were anesthetized after 12 h of fasting for blood sampling from the eyes and then euthanized; brain tissue was removed by decapitation on ice. Fresh blood was allowed to stand for 1 h at room temperature and then centrifuged at 4 °C for 15 min at 3000 r/min to precipitate the blood cells, and the supernatant was stored at −80 °C. The colonic contents were collected in an ultra-low temperature refrigerator at −80 °C for analysis. To be measured, the heart, liver, spleen, kidney, and brain organ indices were calculated as follows: tissue index = tissue weight/body weight × 100%.

### 2.5. Measurement of Oxidation-Related Biomarkers and Inflammatory Cytokines

The whole right part of the brain tissue sample was weighed and homogenized with cold phosphate buffer saline (PBS) (PH7.4) at a weight-to-volume ratio of 1:9 g/mL, and the supernatant was centrifuged (8000× *g*, 4 °C, 10 min) for further analysis. The total antioxidant capacity (T-AOC) content and total superoxide dismutase (T-SOD) activity in serum, tissue protein amount concentration, malondialdehyde (MDA) content, T-AOC content and T-SOD activity in liver tissue, tissue protein amount concentration, and Glutathione peroxidase (GSH-Px) activity in brain tissue were assayed according to the instructions of the kit (Nanjing Jiancheng Institute of Biological Engineering, Nanjing, China). Serum levels of interleukin-10 (IL-10) and tumor necrosis factor-α (TNF-α) were measured using enzyme-linked immunoassay kits IL-10 and TNF-α kits (Shanghai Jianglai Biotechnology Co., Ltd., Shanghai, China), respectively, according to the manufacturer’s instructions.

### 2.6. Brain Tissue Section Observation

Having been dissected and weighed, the left side of the mouse brain was fixed in 4% paraformaldehyde (Hebei Bohai Biological Engineering Development Co., Ltd., Shijiazhuang, China) for more than 24 h. After dehydration, the sample was embedded in paraffin wax, sectioned with a paraffin slicer (Leica, Wetzlar, Germany), spread in a water bath, stained with HE, sealed with neutral resin, and placed under an inverted microscope (400×) The morphological features of the hippocampal region revealed by HE staining were observed.

### 2.7. Investigation of Gut Microbiota

#### 2.7.1. Extraction of Mouse Fecal DNA

Bacterial DNA was extracted from fecal samples by using a fecal genomic DNA extraction kit (Solarbio, Beijing, China) on the manufacturer’s recommendations. Nucleic acids were obtained from 70 mg of fecal samples and eluted in 60 µL of elution buffer. The concentration of DNA was measured by using an infinite M200 pro continuous wavelength multifunctional microporous detector (Tecan, Männedorf, Switzerland). The extracted DNA samples were stored at −80 °C.

#### 2.7.2. Mouse Fecal DNA PCR Reaction

A total of five bacteria were quantified from each fecal DNA sample using real-time fluorescent quantitative polymerase chain reaction (PCR). DNA amplification and detection were performed by using a Roche LightCycler 96 real-time PCR instrument (Roche Diagnostics Co., Ltd., Basel, Switzerland). Samples were routinely analyzed using SYBR Green qPCR Master Mix in a total volume of 20 µL. Each reaction consisted of 2 µL template DNA, 7 µL ddH2O, 10.0 µL of 2 × SYBR qPCR Master Mix (Vazyme Biotech Co., Ltd., Nanjing, China), 0.5 µL of primer 1, and primer 2 (10 µM), and real-time PCR conditions included an initial denaturation step of 95 °C for 5 min, followed by 40 cycles of denaturation at 95 °C for 30 s, primer optimal annealing temperature annealing (Table 1) for 30 s, extension at 72 °C for 30 s, and extension at 72 °C for another 8 min. At the end of the PCR assay, a dissociation curve analysis was performed to check for nonspecific products and/or SYBR Green probe contamination. The relative quantification method was used, and the relative expression of the strains was calculated using the formula as follows:

Relative expression level = 2^−{(Ct value of target gene to be tested − Ct value of internal reference gene to be tested) − (Ct value of control target gene − Ct value of control internal reference gene)}^ (1)

### 2.8. Statistical Analysis of Date

SPSS (version 26.0; SPSS Inc., Chicago, IL, USA) was used to statistically analyze the experimental data, and the experimental data were expressed as $\bar{x}$ ± s. Comparisons between two groups were made by using independent samples *t*-test, comparisons between multiple groups were made using one-way ANOVA and Duncan’s test, and *p* < 0.05 indicated that the data were statistically significant and were plotted using GraphPad Prism 8.0 (GraphPad Software, San Diego, CA, USA).

## 3. Results

### 3.1. Effect of CDFC on Body Weight and Organ Coefficients in Aging Mice

As intervention time becomes longer, the overall body weight of mice in the SD group showed a decreasing trend, indicating that the body weight of aging mice decreased with the aging of the organism (Table 2). The body weight of mice in LDF and MDF groups increased compared with pre-intervention, and the body weight of mice in the HDF group was basically stable throughout the intervention process. Compared with the NDF group, the coefficients of all organs in the three CDFC groups were improved (Table 3). The coefficients of heart, spleen, and brain tissues were significantly higher in the dose groups of mice in the MDF and HDF groups (*p* < 0.05), which were 13.33%, 33.33%, and 23.65% in the MDF group and 11.11%, 30.00%, and 21.62% in the HDF group, respectively. Additionally, the organ coefficients of heart, spleen, and brain tissues were improved to some extent in the three CDFC groups compared with the SD group, but there was no significant difference (*p* > 0.05).

### 3.2. Effects of CDFC on Anxiety-like Behavior in Field Tests

**Figure 1:**
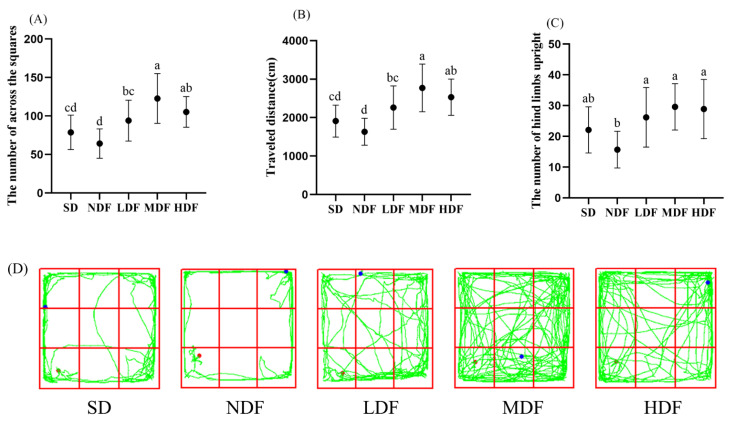
Effect of CDFC on exploration ability of aging mice: (**A**) the number of across the squares; (**B**) traveled distance; (**C**) the number of hind limbs upright. Different superscript letters denote a significant difference in the same index (*p* < 0.05). (**D**) representative traces of mice in different groups. (the green line is the trajectory; the red dot is the starting point; the blue dot is the ending).

The open field test has been widely used to assess motor activity and exploratory behavior in aging mice [23,24]. The number of squares traversed, total motor distance, and the number of hind limbs upright significantly increased in the three CDFC groups of mice compared to the NDF group (*p* < 0.05) (Figure 1). Compared with the NDF group, the number of squares traversed, the total distance of movement, and the number of hind limbs upright increased by 91.28%, 69.83%, and 88.53%, respectively, in the MDF group. However, there was no significant difference in the number of squares traversed, the total distance of movement, and the number of hind limbs upright in the NDF group compared with the SD group. Combining the movement routes of mice in the open field of each group shows (Figure 1D) that mice in the NDF and SD groups had a narrow range of movement, hardly moving in the center of the open field and tending to move around the open field. In contrast, mice in the CDFC group had a wide range of movement in the open field and tended to move more in the center of the field, and mice in the MDF group performed better. This suggests that CDFC may increase the locomotor ability of aging mice.

### 3.3. Effect of CDFC on the Morris Water Maze Test

**Figure 2:**
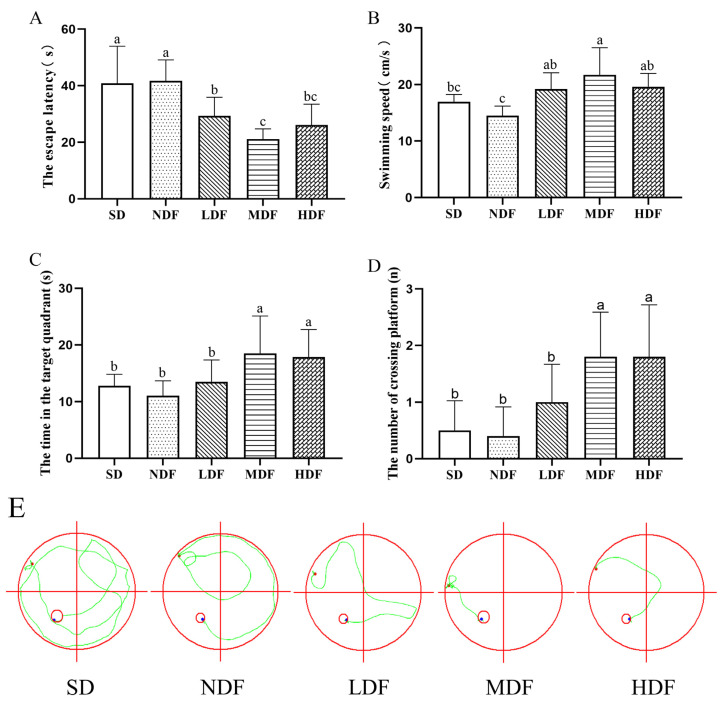
The spatial learning and memory capacity of the mice were tested and analyzed using the Morris water maze over 4 days (*n* = 10): (**A**) Escape latency; (**B**) swimming speed; (**C**) time spent in target quadrant; (**D**) the number of times crossing the platform. Different superscript letters denote a significant difference in the same index (*p* < 0.05). (**E**) the swimming trajectory of the different groups during the 60 s probe (the red circle is the exploration area; the green line is the trajectory; the red dot is the starting point; the blue dot is the ending).

To assess the effect of CDFC on improving spatial learning and memory abilities in aging mice, we performed a hidden platform acquisition training (Table 4) and a detection test (Figure 2).

The changes in escape latency of each group of mice during the four days of the positioning navigation test are shown in Table 4. In the first day of the positioning navigation test, there was no significant difference in the escape latency between the groups. After four consecutive days of training, the escape latencies of all three CDFC groups were significantly reduced, indicating that the spatial learning and memory of mice in the dietary fiber complex group improved. However, the escape latency of the SD group of mice did not significantly decrease with training throughout the experiment (*p* < 0.05), indicating a relatively severe degradation of learning and memory ability in aging mice. Similarly, there was no significant change in escape latency in the NDF group of mice. However, the escape latency of the three CDFC groups was significantly reduced (*p* < 0.05) with the increase in training time, and the escape latency of the three CDFC groups was reduced by 40.73% (*p* < 0.01), 50.07 % (*p* < 0.01), and 47.39 % (*p* < 0.01) on the fourth day compared with the first day, which was significantly lower than that of the SD and NDF groups, respectively. Moreover, there was a significant difference in escape latency from the second day onward in both the MDF and HDF groups compared to the first day.

**Figure 3:**
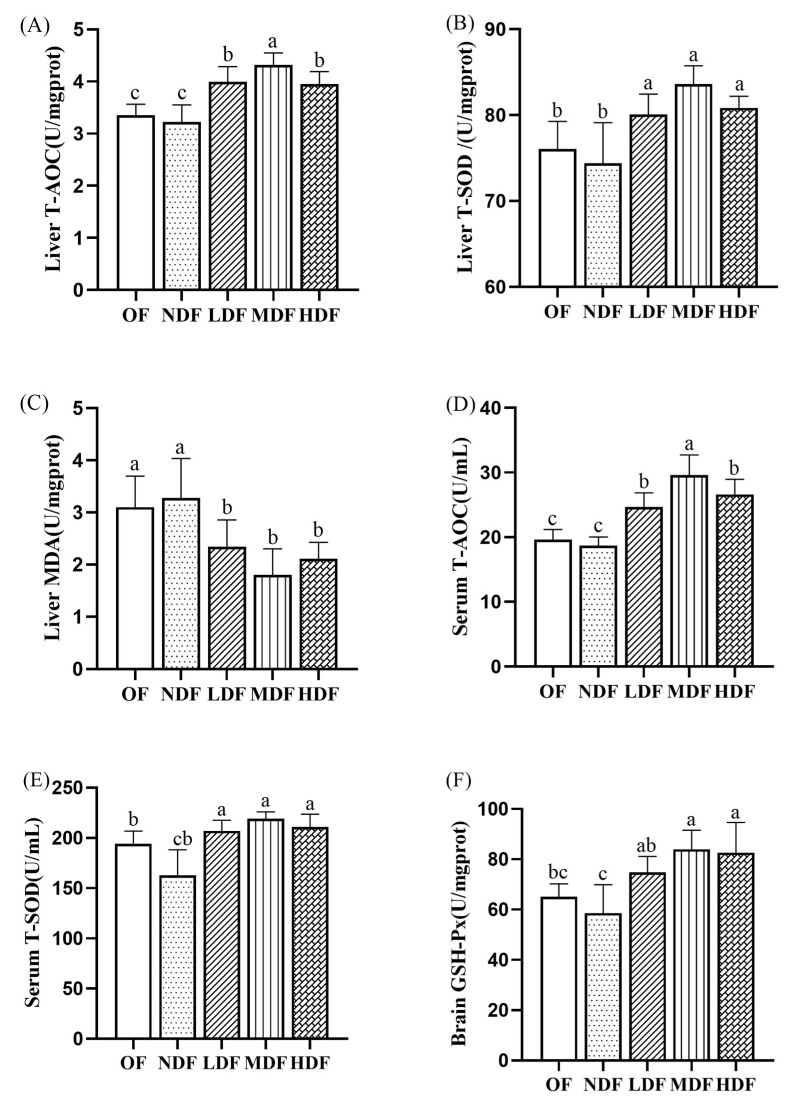
Effect of CDFC on T-AOC and T-SOD activity in mouse liver tissues (**A**,**B**) and serum (**D**,**E**); MDA content in liver (**C**) and GSH-Px level in brain tissue (**F**). Different superscript letters denote a significant difference in the same index (*p* < 0.05).

In the spatial exploration experiment, after 8 weeks of intervention, the mice in the dietary fiber complex group could find the platform quickly compared to the SD group (Figure 2E). Additionally, the escape latency was significantly shorter in all three CDFC groups compared to the NDF group (*p* < 0.05), in which the escape latency was 49.44% shorter in the MDF group (*p* < 0.05). Swimming speed is also an important indicator of aging in the mice. As can be seen in Figure 3B, the swimming speed of the CDFC group was significantly higher than that of the NDF group (*p* < 0.05), and among them, the mice in the MDF group swam the fastest, with a significant increase of 49.78% compared with the NDF group (*p* < 0.05). The residence time and the number of platforms traversed by the mice in the target quadrant correlated with their memory of the location of the platform during training. Figure 2C,D shows that the mice in each group showed more consistent results in the dwell time and number of platforms traversed in the target quadrant, with all three CDFC groups showing an increase in the dwell time and number of platforms traversed in the target quadrant compared to the SD group, with the MDF group showing the best performance in comparison, with a 67.55% (*p* < 0.05) and 350% (*p* < 0.05). In contrast, there were no significant differences between the NDF and SD groups in escape latency, swimming speed, target quadrant dwell time, and the number of platforms traversed.

### 3.4. Effect of CDFC on Antioxidant Capacity

As shown in Figure 3, after dietary intervention, the T-AOC and T-SOD activity in liver tissues and serum significantly increased in the three CDFC groups compared with the NDF group (*p* < 0.05), and especially in the MDF group, where T-AOC and T-SOD activity increased by 33.96% and 12.40% in liver tissues and 58.29% and 34.48% in serum, respectively. Compared with the SD group, the NDF group showed lower T-AOC and T-SOD activity in liver tissues and serum, but neither was significantly different. MDA is a key indicator of lipid peroxidation induced by oxidative damage; therefore, maintaining the antioxidant defense system seems to require the control of lipid peroxidation. The MDA content was lower in all three CDFC groups than in the SD group, with the greatest reduction in the MDF group, which was significantly lower by 45.02% (*p* < 0.05). In contrast, the MDA content in the liver tissue of mice in the NDF group was higher than that in the SD group, but the difference was not significant. GSH-PX is an important antioxidant peptide widely present in the brain, reflecting the antioxidant properties of cells [25]. Compared with the SD group, the GSH-Px content in the brain tissue of mice in the NDF group was slightly lower, but there was no significant difference; the GSH-Px activity in the brain tissue of mice in all three CDFC groups increased to some extent, with the most significant increase in the MDF group, which increased by 28.79% (*p* < 0.01). Taken together, these results show that CDFC can slow oxidative stress and delay aging.

### 3.5. Effect of CDFC on Serum Inflammatory Markers in Aging Mice

**Figure 4:**
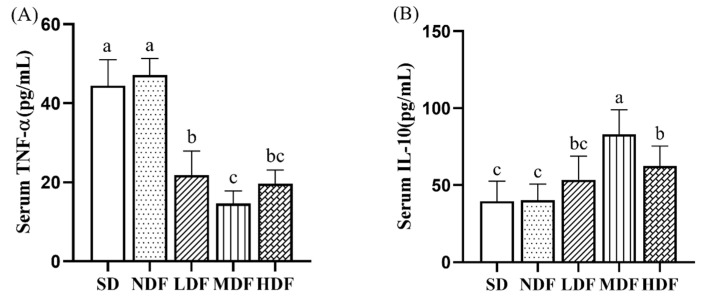
Inflammatory cytokine level in serum in different group after 8 weeks of treatment: TNF-α (**A**) and IL-10 (**B**). Different superscript letters denote a significant difference in the same index (*p* < 0.05).

To assess the effect of CDFC on inflammation levels in aging mice, we measured the serum concentrations of the pro-inflammatory factor TNF-α and the anti-inflammatory factor IL-10 (Figure 4). After 8 weeks of intervention, serum TNF-α concentrations were significantly lower (*p* < 0.01) in mice from the three CDFC groups compared to the SD group by 50.88%, 67.02%, and 55.85%, respectively, while serum IL-10 concentrations were increased in mice from the three CDFC groups by 34.96% (*p* > 0.05), 109.43% (*p* < 0.01), and 57.81% (*p* < 0.05), respectively. The results suggest that CDFC intervention increases the levels of anti-inflammatory factors and decreases the levels of pro-inflammatory factors in aging mice. In addition, it is important to note that the results show that the MDF and HDF groups have a better improvement effect.

### 3.6. Protective Effect of CDFC on the Hippocampal Region of Aging Mice

**Figure 5:**
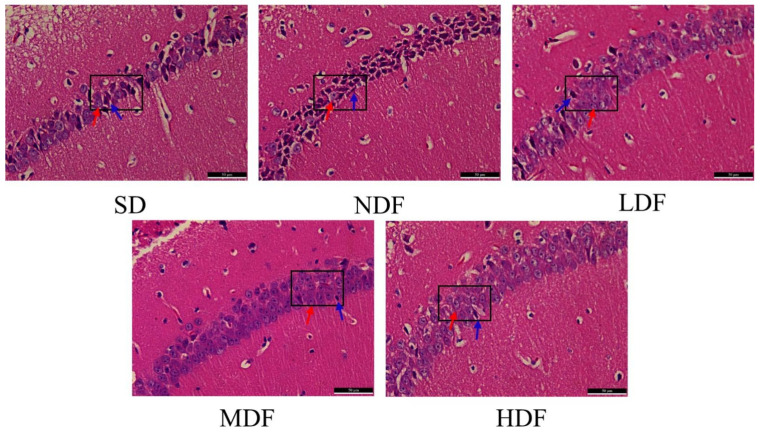
Representative images of HE staining in the brain (magnification, 400×); red arrows indicate normal cells, blue arrows indicate crinkled cells.

In order to investigate whether CDFC has a protective effect on the hippocampal region of aging mice, hematoxylin and eosin (H&E) staining was used to assess the extent of damage in the hippocampal CA1 region of aging mice (Figure 5). In the LDF group, the number of layers and the number of cone cells in the hippocampal CA1 area of mice increased compared with the SD group, where a small number of nuclei were fixed, and apoptosis was improved. In the NDF group, compared with the SD group, the cone cells in the hippocampal CA1 area of mice had increased interstitial space and decreased cytoplasm; most of the cells had solidified deep stained nuclei, and very few cells had normal morphology. This suggests that CDFC also has the effect of preventing damage to the hippocampal region of brain tissue in aging mice.

### 3.7. Perturbation of Fecal Typical Microorganisms by CDFC Intervention

**Figure 6:**
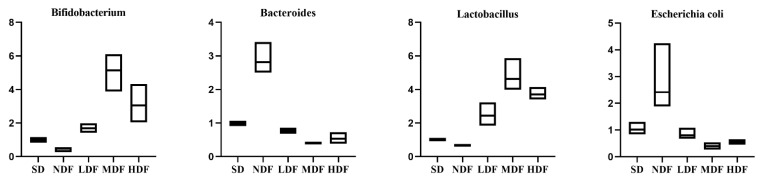
Relative expression of fecal flora in each group of mice.

Total intestinal flora was used as the internal reference gene, and *Escherichia coli*, *Bacteroides*, *Bifidobacterium*, and *Lactobacillus* were the target bacterial genera. The relative fecal expression of the four important bacterial groups in each group of mice after the CDFC intervention is shown in Figure 6. The expression levels of *Bifidobacterium* and *Lactobacillus* significantly increased in all three CDFC groups compared with the SD group (*p* < 0.05), with an increase of 412.94% (*p* < 0.05) and 362.48% (*p* < 0.05) in the MDF group. Expression levels of *Bifidobacterium* and *Lactobacillus* decreased in the NDF group compared with the SD group, by 52.16% (*p* > 0.05) and 5.25% ((*p* > 0.05)). In addition, the expression levels of *Bifidobacterium* and *Escherichia coli* were significantly decreased in the three DFC groups compared with the SD group (*p* < 0.05). Among them, the MDF group showed the best performance with a noticeable decrease of 59.10% (*p* < 0.01) and 61.52% (*p* < 0.05), respectively.

### 3.8. Interaction of Fecal Microbes with Behavioral, Antioxidant Indicators and Inflammatory Markers

**Figure 7:**
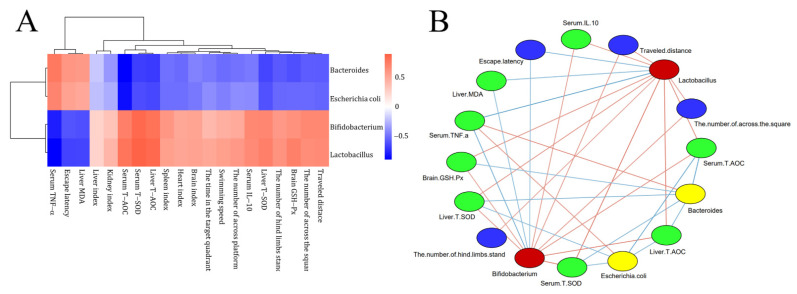
Correlation of gut flora with aging-related parameters: (**A**) Heat map of correlations between key microorganisms and aging-related parameters; (**B**) association network of key microorganisms and aging-related parameters. Red nodes: key microorganisms with increased dietary fiber complex intervention; yellow nodes: key microorganisms with decreased dietary fiber complex intervention; blue nodes: behavioral indicators; green nodes: biochemical parameters. Red lines indicate positive correlations and blue lines indicate negative correlations. Color shades indicate correlation strength. Only valid edges were plotted in the network (|r| > 0.6, *p* < 0.05).

To characterize the anti-aging effect of CDFC, the correlation between the microorganisms reflected by the CDFC intervention and each aging-related parameter was explored based on correlation coefficients (Figure 7). Five organ indices were positively correlated with *Bifidobacterium* and *Lactobacillus* abundance, and negatively correlated with *Bacteroides* and *Escherichia coli*. All six behavioral indices were positively correlated with *Bifidobacterium* and *Lactobacillus* abundance, except for escape latency. Liver levels of MDA and blood levels of TNF-α were positively correlated with *Bifidobacterium* and *Escherichia coli*. Liver levels of GXH-Px, serum levels of T-SOD, T-AOC and IL-10, and liver levels of T-SOD and T-AOC were positively correlated with *Bifidobacterium* and *Lactobacillus*.

## 4. Discussion

In this study, we found that longevity dietary pattern-mediated CDFC has a bidirectional modulatory effect on body weight in naturally aging mice. Body weight is closely related to healthy living in the elderly [26]. Both obesity and underweight can have adverse health effects [27]. Previous studies have shown that aging mice suffer from significant memory loss, weight loss, and decreased organ indices [28,29]. In the present study, we found that mice fed standard chow significantly lost body weight during aging, and CDFC inhibited aging-induced weight loss. Additionally, CDFC attenuated the weight gain caused by dietary fiber-free diets. In contrast, one study found that oligofructose significantly reduced diet-induced obesity and that the combination of oligofructose and resistant starch, or β-glucan, did not reduce obesity [10]. The different results between these studies may be related to the type and amount of dietary fiber as well as individual differences. In addition, in this study, mice fed standard chow exhibited atrophy of brain, spleen, and heart, while CDFC improved the corresponding organ indices. Notably, the NDF group also exhibited lower organ indices, which may be related to their higher body weight. This suggests that CDFC can delay organ degeneration and improve organ immunity in mice, and it has a significant anti-aging effect.

Longevity dietary pattern-mediated CDFC has good maintenance and improvement of cognitive learning and exploratory abilities in naturally aging mice. Aging is an inevitable and complex biological process that is often accompanied by a decline in cognition, exploratory abilities, and learning memory capacity [30]. In this study, the Morris water maze test was used to study the spatial memory and learning abilities of aging mice. In aging mice fed CDFC, escape latency was significantly reduced in all, while swimming speed, target quadrant dwell time, and number of platforms traversed were significantly increased, and they performed better than mice fed regular chow. These results also reflect the anti-aging effect of CDFC in mice. It has been suggested that dietary fiber deficiency may affect the expression of CaMKII and its related synaptic proteins and thus lead to learning memory and cognitive dysfunction [31]. Therefore, the results of the present study may be related to this.

Oxidative stress-induced organ damage can be identified and analyzed through histopathological observations. The hippocampus is an important brain region associated with learning memory function, of which CA1 region is closely related to spatial location and learning memory and is most susceptible to aging. HE staining was used to evaluate the effect of CDFC on hippocampal neuronal cells in aging mice. Histopathological analysis of hippocampal region in SD and NDF group mice exhibited significant tissue damage characterized by necrotic cells, and the morphology and number of neuronal cells in hippocampal region in CDFC group mice were significantly improved; this attenuated symptom of tissue damage indicated that CDFC could protect brain tissue by reducing oxidative stress. At the same time, the reduction in the number of hippocampal neuronal cells was shown to affect the formation of dendritic spines, which in turn leads to changes in synaptic plasticity [32], thus affecting learning and memory abilities.

In addition, cognitive loss is often accompanied by increased anxiety, which can be found in both rodents and humans [33,34]. In the open field test, the animal’s activity area can effectively reflect the animal’s anxiety level, with longer activity time in the central area indicating a lower level of anxiety [35]. In the present study, mice in the CDFC group showed a significant increase in exploratory behavior and time spent in the central area compared to the SD group. Studies have shown that high dietary fiber intake is associated with a reduced prevalence of anxiety [36]. Dietary fiber can regulate anxiety by promoting the production of short-chain fatty acids that effectively modulate inflammatory factors and neurotransmitter levels [37]. Therefore, the modulation of anxiety by CDFC may be relevant.

In terms of characterization of anti-aging improvement mechanisms, the present study found that CDFC in the longevity dietary pattern was able to increase antioxidant capacity and reduce inflammation levels in naturally aging mice. Previous studies have shown that ROS-induced oxidative stress is one of the key factors that induce age-related changes [29]. After eight weeks of intervention, CDFC significantly increased T-SOD and T-AOC activities in serum and liver tissues and GSH-PX levels in brain tissues of aging mice and also significantly decreased MDA that was produced. T-AOC is an important nonenzymatic antioxidant, and its decreased levels are associated with a reduced resistance of the body’s antioxidant defense system against free radicals [38]. T-SOD is a free radical scavenging enzyme in vivo key enzyme that converts superoxide anion radicals into hydrogen peroxide and oxygen to prevent oxidative stress damage [39]. Studies have shown that dietary fiber contains the presence of some active substances (e.g., polyphenols) [40], which, as key components of dietary fiber, can exert antioxidant effects by complexing metal ions, increasing antioxidant enzyme activity, scavenging free radicals, and regulating the expression of signaling pathways, such as Nrf2/ARE [41,42], which is more consistent with the results obtained in this study. Therefore, CDFC can alleviate aging-induced oxidative damage in the organism and has potential anti-aging effects.

To further determine the anti-aging effect of CDFC, inflammatory markers in the serum of mice in each group were examined in this study. The results revealed that the levels of pro-inflammatory factor TNF-α were significantly lower and the levels of IL-10 were significantly higher in the CDFC group of mice after the intervention compared to SD. Inflammation is an inherent part of the immune system, and changes in blood inflammatory markers can be used to predict the health status and lifespan of the organism [43]. Numerous studies have shown that diets rich in dietary fiber significantly reduce systemic inflammation, which is an important factor in the effects of aging [44,45]. On the one hand, short-chain fatty acids produced by fermentation of dietary fiber attenuate the gene expression levels of the pro-inflammatory cytokines IL-1β, TNF, and IL-6 and improve neuroinflammation in aged mice [46]. On the other hand, dietary fiber intake controls postprandial hyperglycemia, reduces oxidative stress, and attenuates the inflammatory response by regulating the NF-kB pathway [47]. Therefore, it is believed that the low inflammation level caused by CDFC may be related to this. Notably, TNF-α is not only an indicator of inflammation but also contributes to morbidity and mortality in old age [48]. Taken together, these results suggest that the anti-aging effect of CDFC is reflected in the maintenance of immune homeostasis.

The aging process is accompanied by changes in the gut microbiota. Longevity dietary pattern-mediated CDFC can alter the expression levels of intestinal microbes in C57BL/6 aging mice. The expression of *Bifidobacterium* and *Lactobacillus* was significantly increased after CDFC intervention. Legume dietary fiber was reported to increase the relative abundance of *Bifidobacterium* in the feces of mice [49]. The intake of dextran resulted in a dose-dependent decrease in *Bacteroides* and an increase in *Lactobacillus* and *Bifidobacterium* [50]. This increase may be due to the ability of *Lactobacillus* and *Bifidobacterium* to digest dietary fiber and other carbohydrates [51]. Furthermore, it has been shown that *Bifidobacterium* and *Lactobacillus* can act as anti-inflammatory molecules through the production of short-chain fatty acids, such as acetic acid and propionic acid in the intestine [52], capable of inhibiting NF-κB activation in host immune cells by binding to G protein-coupled receptors 43 and 41 (GPR43 and GPR41), thereby blocking the inflammatory response and inhibiting the release of TNF-α and IL-6 to aid healthy aging [53]. This was confirmed by the correlation analysis in the present study, i.e., *Bifidobacterium* and *Lactobacillus* were significantly associated with aging-related parameters, such as oxidative parameters and inflammatory factors. In addition, an excessive number of *Escherichia coli* in the intestinal flora disrupts the intestinal barrier and adversely affects the intestinal health of the organism. In the present study, CDFC reduced *Escherichia coli* abundance in mouse feces, suggesting that CDFC could improve host health by reducing pathogenic bacteria levels.

To the best of our knowledge, this study is the first to exclude basal dietary fiber from feed and to investigate the anti-aging effect on the characteristic dietary fiber compound in the longevity dietary pattern. Most of the current studies on dietary fiber have intervened based on standard diets without excluding the effect of the original dietary fiber in the standard diets. In addition, the overall effect of the CDFC combination in this study was better in the MDF group, which suggests that the anti-aging effect of CDFC is not in a dose-dependent relationship, and there may be an optimal, additional value of 15%. This is the first time that such a situation has been found since no relevant studies on the optimal intake dose of compound dietary fiber have been seen; therefore, we will follow up with a more in-depth study on the optimal intake dose of CDFC.

## 5. Conclusions

This study explored the protective effects of CDFC on aging mice under the Guangxi longevity dietary pattern. The results showed that CDFC could have protective effects on aging mice by slowing aging-induced brain neuronal cell and tissue organ damage, improving body immunity, enriching the levels of *Bifidobacterium* and *Lactobacillus*, and decreasing the expression of *Escherichia coli* and *Bacteroides*. These results strongly demonstrate that CDFC mediated by Guangxi longevity dietary pattern has the effect of improving the antioxidant level of the body, reducing the level of inflammation, and delaying aging. The analysis also revealed a bidirectional regulatory effect of CDFC, with a maintenance and an ameliorative effect on aging-induced weight loss and weight gain due to dietary fiber-free diets. In addition, the optimal amount of CDFC addition was deduced from the level of experimental animals to be about 15%. This provides a new perspective for the interpretation of why dietary fiber is beneficial to healthy aging and also provides theoretical support for the mechanism of action of the anti-aging effect presented by the longevity dietary pattern.

## Figures and Tables

**Table 1:** Primer sequences of five species.

Bacteria	Primer Sequence (5′–3′)	Annealing Temperature	Reference
Total intestinal flora	F: 5′-ACTCCTACGGGAGGCAGCAG-3′ R: 5′-ATTACCGCGGCTGCTGG-3′	60 °C	Hélène A.et al. [18]
*Bacteroides*	F: 5′-CTGAACCAGCCAAGTAGCG-3′ R: 5′-CCGCAAACTTTCACAACTGACTTA-3′	62 °C	Yanming W.et al. [19]
*Bifidobacterium*	F: 5′-TCGCGTCCGGTGTGAAAG-3′ R: 5′-CCACATCCAGCATCCAC-3′	58 °C	Jing, W.et al. [20]
*Lactobacillus*	F: 5′-AGCAGTAGGGAATCTTCCA-3′ R: 5′-CACCGCTACACATGGAG-3′	58 °C	Tingting, S.et al. [21]
*Escherichia coli*	F: 5′-GTTAATACCTTTGCTCATTGA-3′ R: 5′-ACCAGGGTATCTTAATCCTGTT-3′	60 °C	Nelson, E. A. et al. [22]

**Table 2:** Effect of CDFC on body weight of aging mice.

Group	Intervention Time/Week				
	0	2	4	6	8
SD	31.18 ± 5.13	29.5 ± 3.98	28.76 ± 3.57 ^b^	29.24 ± 3.6 ^b^	28.78 ± 4.69 ^b^
NDF	30.9 ± 4.34 ^B^	32.85 ± 4.07 ^AB^	32.73 ± 3.83 ^aAB^	33.29 ± 4.06 ^aAB^	35.37 ± 5.08 ^abA^
LDF	30.79 ± 3.00 ^B^	31.93 ± 3.32 ^AB^	31.75 ± 2.76 ^abAB^	32.05 ± 3.14 ^abAB^	34.48 ± 3.51 ^aA^
MDF	31.47 ± 3.37 ^AB^	32.04 ± 3.94 ^AB^	31.36 ± 3.89 ^abB^	31.94 ± 3.75 ^abAB^	34.98 ± 3.41 ^aA^
HDF	31.39 ± 5.78	31.66 ± 5.48	31.05 ± 4.87 ^ab^	31.22 ± 4.89 ^ab^	32.03 ± 5.78 ^a^

In the same column, different superscript lowercase letters indicate significant differences between groups (*p* < 0.05). In the same row, different superscript capital letters indicate significant differences in the same group at different intervention times (*p* < 0.05).

**Table 3:** Effect of CDFC on organ indices of aging mice.

Group	Organ Index				
	Heart	Liver	Spleen	Kidney	Brain
SD	0.46 ± 0.03 ^b^	3.98 ± 0.39	0.34 ± 0.07 ^b^	1.20 ± 0.14	1.59 ± 0.16 ^b^
NDF	0.45 ± 0.03 ^ab^	3.92 ± 0.39	0.30 ± 0.10 ^a^	1.10 ± 0.11	1.48 ± 0.21 ^ab^
LDF	0.48 ± 0.03 ^a^	4.00 ± 0.39	0.37 ± 0.0.08 ^a^	1.22 ± 0.10	1.68 ± 0.22 ^a^
MDF	0.51 ± 0.05 ^a^	4.11 ± 0.40	0.40 ± 0.09 ^a^	1.35 ± 0.31	1.83 ± 0.16 ^a^
HDF	0.50 ± 0.03 ^ab^	4.19 ± 0.37	0.39 ± 0.08 ^ab^	1.28 ± 0.15	1.80 ± 0.22 ^b^

In the same column, different superscript lowercase letters indicate significant differences between groups (*p* < 0.05).

**Table 4:** Effect of hidden-platform acquisition training test for four consecutive days.

Group	Escape Latency(s)			
	Day 1	Day 2	Day 3	Day 4
SD	57.70 ± 4.87	53.74 ± 7.12	51.11 ± 9.64	44.05 ± 14.32 ^a^
NDF	58.07 ± 3.83	54.38 ± 9.14	52.82 ± 9.43	46.90 ± 10.38 ^a^
LDF	57.87 ± 3.88	51.94 ± 9.82	46.95 ± 14.05	34.30 ± 9.86 ^b^**
MDF	57.48 ± 4.29	50.56 ± 6.22 *	42.44 ± 9.96 *	28.70 ± 10.85 ^b^**
HDF	58.05 ± 2.74	51.38 ± 9.58 *	46.19 ± 7.55 *	30.54 ± 9.01 ^b^**

All values are expressed as mean ± standard deviation (*n* = 10). In the same column, different superscript lowercase letters indicate significant differences between groups (*p* < 0.05). Asterisks indicate significant differences (compared with the first day * *p* < 0.05, ** *p* < 0.01).

## Data Availability

The data in this study are available on request from the author.
